# Hierarchical predictive coding in distributed pain circuits

**DOI:** 10.3389/fncir.2023.1073537

**Published:** 2023-03-03

**Authors:** Zhe Sage Chen

**Affiliations:** ^1^Department of Psychiatry, New York University Grossman School of Medicine, New York, NY, United States; ^2^Department of Neuroscience and Physiology, New York University Grossman School of Medicine, New York, NY, United States; ^3^Neuroscience Institute, NYU Grossman School of Medicine, New York, NY, United States; ^4^Department of Biomedical Engineering, NYU Tandon School of Engineering, Brooklyn, NY, United States; ^5^Interdisciplinary Pain Research Program, NYU Langone Health, New York, NY, United States

**Keywords:** hierarchical predictive coding, pain network, cingulate-insula hub, prediction error, active inference, neural oscillations, traveling waves, neurotransmitter

## Abstract

Predictive coding is a computational theory on describing how the brain perceives and acts, which has been widely adopted in sensory processing and motor control. Nociceptive and pain processing involves a large and distributed network of circuits. However, it is still unknown whether this distributed network is completely decentralized or requires networkwide coordination. Multiple lines of evidence from human and animal studies have suggested that the cingulate cortex and insula cortex (cingulate-insula network) are two major hubs in mediating information from sensory afferents and spinothalamic inputs, whereas subregions of cingulate and insula cortices have distinct projections and functional roles. In this mini-review, we propose an updated hierarchical predictive coding framework for pain perception and discuss its related computational, algorithmic, and implementation issues. We suggest active inference as a generalized predictive coding algorithm, and hierarchically organized traveling waves of independent neural oscillations as a plausible brain mechanism to integrate bottom-up and top-down information across distributed pain circuits.

## Introduction

Pain is a dynamic and multi-dimensional experience. Multi-dimensions of pain processing are defined by three independent yet interleaved components—that is, sensory-discriminative, affective-emotional, and cognitive-motivational components (Rainville et al., 1997; Price, 2000; Ploner et al., 2017). Unlike other sensory cortices, there is no “pain cortex”. Instead, a distributed network of cortical-subcortical-brainstem areas (also known as “pain matrix”) is involved in pain processing (Iannetti and Mouraus, 2010; Garcia-Larrea and Peyron, 2013; Mano and Seymour, 2015). In the past decades, advances in electrophysiological recordings, neuroimaging, optogenetics, and neuromodulation have greatly enhanced our capability to dissect neural mechanisms of pain circuits (Mouraux and Iannetti, 2018; Kuner and Kuner, 2021). Because of the distributed nature of pain processing, a holistic, systems-level understanding of how different neural circuits transfer, coordinate, and integrate information still remains elusive. In addition, several computational theories have been proposed in pain studies (see a review in Chen and Wang, 2023), including reinforcement learning and control (Seymour, 2019; Seymour and Mancini, 2020; Mancini et al., 2022; Seymour et al., 2023), and predictive coding (Büchel et al., 2014; Wiech, 2016; Ploner et al., 2017; Jepma et al., 2018).

Predictive coding accommodates a wide class of general ideas of inference from generative models in the brain (Huang and Rao, 2011; Bastos et al., 2012; Aitchison and Lengyel, 2017; Spratling, 2017). As a generative model, the brain receives input data from sensory stimulation, makes statistical assumptions based on the current knowledge of the world, and quickly update the prediction using feedback. Hierarchical predictive coding further generalizes this notion in that the brain uses multiple structures of predictive assumptive models to optimize perception and action (Friston, 2005; Kiebel et al., 2008; Wacongne et al., 2011), providing a more general framework to understand the control hierarchy and distributed information processing.

In this mini-review, we revisit important pain circuits and pathways identified from recent animal and human pain studies, and further review neural evidence that supports predictive coding in the context of pain studies. Although our understanding of individual local neural circuits continues improving, a high-level holistic comprehension is still poor. We then touch on the central question of this article: what is the computational mechanism to integrate information across distributed pain circuits, and how to implement it? Following Marr’s three levels of analysis (Marr, 1982), we discuss these questions at the computational, algorithmic, and implementation levels. Specifically, we propose an updated hierarchical predictive coding framework for pain processing. At the core of this framework, the cingulate cortex and insula cortex play a role of central hub in mediating the information from sensory afferents and spinothalamic inputs. At the algorithmic level, we suggest active inference as generalized predictive coding algorithms to accommodate the pain perception-action cycle. At the implementation level, we suggest that hierarchically organized traveling waves of independent neural oscillations serve as a plausible brain mechanism to integrate bottom-up and top-down information across distributed pain circuits. While several components of the proposed theory remain largely speculated, they can be experimentally tested with the advances in large-scale neural recordings and causal manipulation tools.

## The pain network and cingulate-insula hub

Numerous human neuroimaging data have shown that a large distributed network of cortical and subcortical regions collectively processes and integrates nociceptive signals to give rise to an overall pain experience. The mammalian pain system consists of ascending and descending pathways, including the peripheral nerves, spinal cord, and cerebral cortex. There are two major ascending pain pathways that are anatomically and functionally separable (Price, 2000; Bushnell et al., 2013; Vanneste and De Ridder, 2021). The medial pain pathway involves the dorsal anterior cingulate cortex (dACC) and anterior insula cortex (AIC) as the main nodes, whereas the lateral pain pathway involves somatosensory cortex as the main node. Furthermore, the descending pain inhibitory pathway involves rostral and pregenual anterior cingulate cortex (pgACC), the periaqueductal gray (PAG), hypothalamus, and rostral ventromedial medulla (RVM). Several reviews have discussed these pain pathways in detail (Millian, 2002; Fields, 2004; Vogt, 2005). Together, the pain network of cortical, subcortical, and brainstem structures contribute to various sensory, cognitive, affective, and psychophysiological processes in pain perception and regulation (Tracey and Mantyh, 2007; Costigan et al., 2009; Legrain et al., 2011; Peirs and Seal, 2016; Tan and Kuner, 2021). For the reasons explained below, we suggest that the cingulate cortex and insula cortex jointly form a “cingulate-insula hub” for coordinating information in distributed pain processing.

**Figure 1:**
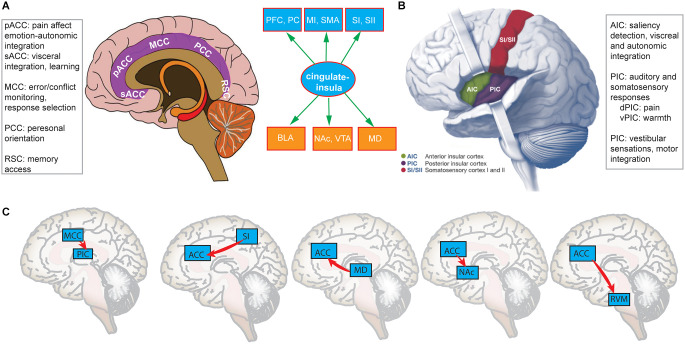
**(A)** A schematic illustration of the cingulate cortex that contains four subdivisions: ACC (including pACC and sACC subregions), MCC, PCC, and RSC, each having distinct functions. (**B**) A schematic illustration of AIC and PIC and their distinct functions. Between panels **(A)** and **(B)** shows a cingulate-insula hub that bridges both cortical areas in ascending pathways and subcortical areas in descending pathways. (**C**) Optogenetically identified cortico-cortical, cortico-subcortical, and cortico-spinal projections originated from the cingulate-insular hub [Panel **(B)** was modified with permission from Chen et al., 2021].

The cingulate cortex includes the entire cingulate gyrus that contains the anterior cingulate cortex (ACC), posterior cingulate cortex (PCC), midcingulate cortex (MCC), and retrosplenial cortex (RSC; Vogt, 2005; Shackman et al., 2011; Nevian, 2017). Notably, the primate medial prefrontal cortex (mPFC) is often referred to as the ACC in rodents (Laubach et al., 2018; van Heukelum et al., 2020), which sometimes cause confusion in terminology because the terms “mPFC” and “ACC” have been used interchangeably in rodent research (Francis-Oliveira et al., 2022). The ACC is a large, heterogeneous region, which also consists of multiple subdivisions that support a wide range of functions (Figure 1A). Generally, the ACC can be divided anatomically based on cognitive (dorsal part) and emotional (ventral part) components. The dorsal ACC is connected with the PFC, parietal cortex (PC), and the motor system (e.g., supplemental motor area, SMA), making it a central station for processing bottom-up and top-down information and assigning appropriate control to other brain areas (Shenhav et al., 2016). In contrast, the ventral ACC is connected with the amygdala, nucleus accumbens (NAc), hypothalamus, and AIC, and is implicated in assessing the salience of emotion and motivational information (Allman et al., 2001). Furthermore, the rostral ACC (rACC) is ideally positioned between limbic and cortical structures to integrate emotion and cognition (Mohanty et al., 2007; Tang et al., 2019), and is strongly connected to the basolateral amygdala (BLA). In the primate brain, the ACC is also the region with the highest time constant that is useful for temporal integration (Murray et al., 2014). The MCC has distinct representations of pain from the ACC, and is more involved in response selection (such as conflict monitoring, approach-avoidance) through the projections to spinal cord and motor cortices (Vogt, 2005).

The insula cortex contains multiple subregions: anterior insula cortex (AIC), mid-insula cortex (MIC), and posterior insula cortex (PIC; Figure 1B). Different subdivisions of the insula have been implicated in a wide range of functions in sensory and affective processing (Craig, 2009; Segerdahl et al., 2015; Namkung et al., 2017; Bastuji et al., 2018). The anatomic location of the insula is also unique. The AIC is connected with the anterior cingulate, frontal, orbitofrontal, and anterior temporal areas, and is responsible for the integration of autonomic and visceral information (Uddin et al., 2017). There is strong structural and functional connectivity between the AIC and ACC (Qadir et al., 2018). The PIC is connected with the posterior temporal, parietal, and sensorimotor areas, and is more responsible for somatosensory, vestibular, and motor integration. Between the AIC and PIC, the MIC is considered as a “transitional area” that shares similar features of both subdivisions (Uddin et al., 2017). There is a differential structural and resting-state connectivity for the anterior, mid, and posterior insula with other pain-related brain regions, supporting their different functional profiles in pain processing (Wiech et al., 2014). Independent of pain research, the insula has already been suggested as a central hub in cognitive control for four key roles (Menon and Uddin, 2010): (i) bottom-up detection of salient events; (ii) integrating cortical-subcortical information to modulate brain or autonomic reactivity to salient stimuli; (iii) switching between different networks (such as somatosensory vs. emotional) to access the brain resources; and (iv) strong functional coupling between with the ACC that facilitates rapid access to the motor system. In pain research, the AIC and MCC also play a role of “salience network” that integrates information about the significance of an impending stimulation into perceptual decision-making for pain anticipation (Wiech et al., 2010).

In the human neuroimaging literature, it has been shown that the ACC and AIC in the ascending medial pain pathway are important for perceiving pain intensity (Favilla et al., 2014). In real-time fMRI neurofeedback on pain, the ACC and AIC are both effective targets to down-regulate the BOLD (blood oxygenation level dependent) activation during feedback, correlating with a decrease in pain rating (Emmert et al., 2014). Furthermore, the functional connectivity between the AIC and MCC changed as a function of stimulus-contextual information (Wiech et al., 2010) or a function of the subjective motivational urge to escape pain through movement (Perini et al., 2020). In a recent study, participants performed a task that involved predicting a painful or nonpainful stimulus based on the administration of another painful or nonpainful stimulus. It was found that predicted pain increased activations in the ACC, MCC, AIC, and MIC; the MCC activation showed a direct relationship with the motor output, whereas the insula activation was modulated by potential action consequences (Koppel et al., 2023). However, because of the limited spatiotemporal resolution, human neuroimaging only provides correlational findings. Fortunately, innovations in optogenetics have enabled us to causally identify many *direct* cortico-cortical, cortico-subcortical, and cortico-spinal ascending/descending pain pathways originated from the cingulate-insula hub (Figure 1C). There is a direct pathway from the primary somatosensory cortex (SI) to the rACC, chronic pain recruits more pain-modulated ACC neurons through enhancing the cortico-cortical projection, whereas optogenetic modulation of this projection regulates aversive responses to pain (Singh et al., 2020). In the bidirectional pathway between the mediodorsal (MD) thalamus and the ACC, reducing the excitation of ACC neurons to MD inputs causes excitation/inhibition (E/I) imbalance in pain; activating MD inputs elicits pain-related aversion, whereas inhibition of subcortically-projecting ACC neurons reproduces the same effect (Meda et al., 2019). In the descending pathway from the ACC to RVM, direct cortico-spinal modulation by optogenetics causes behavioral pain sensitization, whereas inhibiting the same projection induces an analgesic effect (Chen et al., 2018). The direct projection from the ACC to NAc controls the social transfer of pain and analgesia; optogenetic activation of the ACC→NAc projection selectively enhances pain empathy, yet the ACC→BLA projection is involved in the social transfer of fear (Smith M. L. et al., 2021). The ACC also directly projects to the ventral tegmental area (VTA). It was found that the ACC→NAc/VTA projection mediates aversion of chronic pain, in which the ACC activates NAc D2-type medium spiny neurons, and inhibits the VTA by activating GABAergic neurons after chronic pain treatment (Gao et al., 2020). There is also an afferent projection from the MCC to the PIC. Although the MCC does not mediate acute pain sensation and pain affect, it can regulate nociceptive hypersensitivity (Tan et al., 2017). In addition, glutamatergic projection from the insula to the BLA is critical for the formation of observational pain; selective activation or inhibition of the insula→BLA projection strengthens or weakens the pain intensity, respectively (Zhang et al., 2022). The PIC–>BLA pathway also mediates aversive state processing and anxiety-related behaviors (Gehrlach et al., 2019). Together, these human and animal studies support the role of cingulate-insula hub in regulating pain perception, pain affect, pain analgesia, and pain empathy. Based on this reasoning, a theory for chronic pain was proposed; that is, chronic pain is caused by imbalance between bottom-up pain input and top-down pain suppression (Vanneste and De Ridder, 2021). Specifically, chronic pain subjects are characterized by an abnormal ratio between the somatosensory cortex (gamma power) + dACC (beta power), and pgACC (theta power); the somatosensory cortex and dACC account for the ascending pathway, whereas pgACC is involved in the descending pathway.

If we accept the “cingulate-insula hub” premise, the next question of our central discussion is: what is the underlying computational mechanism and how to implement it? In the following section, we provide several theoretical arguments for the “what” and “how” questions separately.

## Hierarchical predictive coding

We follow a similar analogy of Marr’s analysis and first formulate the problem mathematically (“computational level”), then describe how the identified computational problem can be solved (“algorithmic level”), and finally describe the neural implementation in which computation may be performed (“implementation level”).

**Figure 2:**
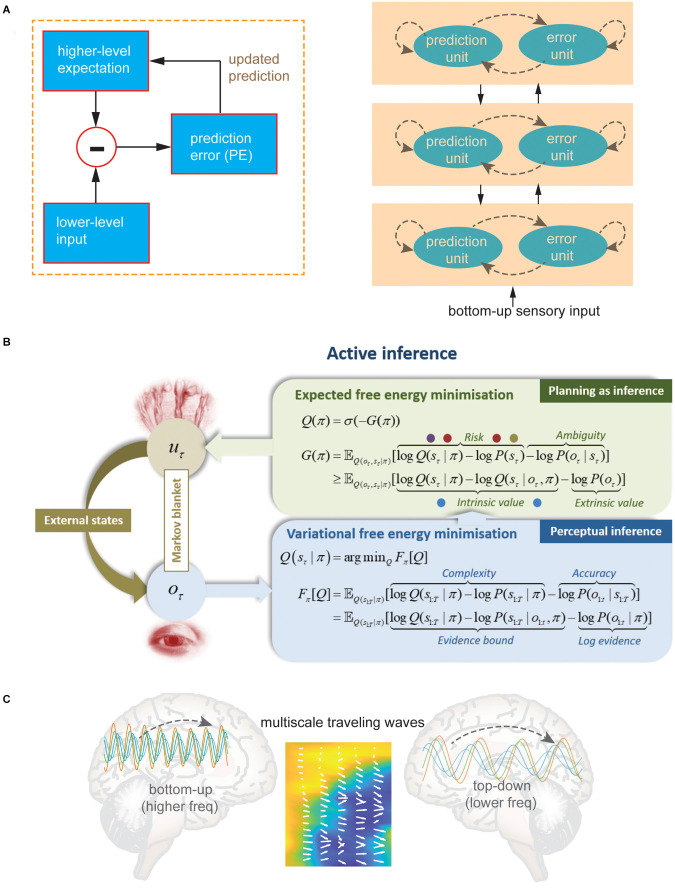
**(A)** A schematic illustration of hierarchical predictive coding. (**B**) Algorithmic description of hierarchical active inference for perception and action (planning). (**C**) Hierarchical organized traveling waves (illustrated by heatmap overlaid by vector fields) orchestrate and integrate bottom-up and top-down information across distributed brain circuits, where bottom-up signaling is represented by higher frequencies and top-down signaling is represented by lower frequencies [Panel **(B)** was modified with permission from Da Costa et al. (2020)].

The core of computational level is predictive coding. Predictive coding theories assume that the brain or individual neural circuit implements inference and predictions using a known (or at least partially known) generative model. Briefly, the local neural circuit receives bottom-up (e.g., nociceptive, sensory, proprioceptive) signals, makes statistical predictions based on the generative model, computes the prediction error (PEs) by comparing top-down signals (e.g., expectation and anticipation), and further updates the model using PEs for subsequent prediction (Figure 2A). Mathematically, it can be simplified by an equation:


Prediction = input + gain × PE

The PE represents a “surprise” signal, and the gain is characterized by the precision of surprise signal. In this equation, the gain modulates the magnitude of PE signals. A small PE or small gain leads to a small correction; in contrast, a large PE or gain leads to a large correction. To illustrate this concept, let us assume that a prediction unit tries to integrate information from a bottom-up unit *x*_1_ and a top-down unit *x*_2_, which carry their own precision parameters $\xi_{1}=\frac{1}{\sigma_{1}^{2}}$ and $\xi_{2}=\frac{1}{\sigma_{2}^{2}}$, respectively. The prediction unit computes a new prediction update as follows


$$x=x_{1}+\frac{\xi_{2}}{\xi_{1}+\xi_{2}}(x_{2}-x_{1})$$

where the relative precision $\frac{\xi_{2}}{\xi_{2}+\xi_{1}}$ defines the gain parameter, and (*x*_2_ − *x*_1_) represents the PE. If we let $\xi =\xi_{1}+\xi_{2}$ denote the new precision, and new prediction is a weighted sum of two inputs, with each weighted by the respective precision parameter: $x=\frac{\xi_{1}}{\xi }x_{1}+\frac{\xi_{2}}{\xi }x_{2}$, then predictive coding will be exactly equivalent to Bayesian integration.

In the context of inference for pain, PEs and predictions may be computed at local pain circuits during various stages of pain processing. Neural communications are possibly manifested in neural oscillations. During early pain processing, inbound nociceptive and other sensory signals may drive the computation (such that $x\approx x_{1}$ in the previous simple example), mostly through the lateral pain pathway. At the later stage, due to the feedback from higher-order areas, top-down signals propagating through other cortical areas may dominate the computation with or without *x*_1_. At the pain-evoked cortical activation level, the activation occurs sooner in the somatosensory cortex than the ACC (Ploner et al., 2002; Xiao et al., 2019).

According to hierarchical predictive coding models (Friston, 2005; Kiebel et al., 2008; Wacongne et al., 2011), distributed pain circuits shall constantly generate predictions of incoming stimuli at multiple levels of processing. Accordingly, a hierarchy of predictions and PEs will be computed at different levels of pain processing along the ascending/descending pathways (Figure 2B). Signals descending the hierarchy via backward connections between brain areas are attributed with conveying predictions, whereas signals ascending the hierarchy propagate the PEs (Friston, 2008). We propose that the cingulate-insula hub can mediate these interareal communications.

### Representations of prediction and prediction error in the ACC-insula hub

First, the ACC has also been long implicated in encoding PE and surprise signals (Brown and Braver, 2005; Hyman et al., 2017; Alexander and Brown, 2019). In human neuroimaging, the ACC has also been known to play a role in monitoring error or conflicts and generating confidence-weighted error signals for cognitive control (Carter et al., 1998), which can be viewed as a weighted PE. This allows the ACC to register “negative surprise” and determine the expected value of control under various circumstances (Raison, 2015). The ACC is connected to the VTA, a dopaminergic region important for motivation and feedback processing. A recent report showed that elevated 4 Hz ACC→VTA signaling is associated with anticipatory decision making (“prediction”), whereas error-related feedback integration is associated with increased VTA→ACC signaling (“PE”), which is also predictive of subsequent choice adaptation (Elston et al., 2019). Recently, it has been suggested that the ACC may engage in a top-down prediction in pain perception through alpha/beta oscillations (Song et al., 2021).

The insula has played a central role in predictive coding, supported by a series of human neuroimaging pain studies (Geuter et al., 2017; Fazeli and Buchel, 2018; Strube et al., 2021; Horing and Büchel, 2022). Notably, the AIC and PIC have slightly different functional roles in predictive coding. While the AIC response to cued pain stimuli matches the PE from the predictive coding model (Geuter et al., 2017), the dorsal PIC directly encodes the stimulus intensity and expectations (Fazeli and Buchel, 2018). In a cross-modality (pain vs. sound) pain study, it has been reported that the AIC response correlates with unsigned intensity PEs as a modality-unspecific aversive surprise signal, whereas dorsal PIC encodes the modality-specific signed intensity PE (Horing and Büchel, 2022). Importantly, pain processing in AIC is modulated by both prediction and action, suggesting its role in mediating pain anticipation (Koppel et al., 2023). Together, the AIC and PIC provide a neural mechanism for predictive coding, and aberrant pain processing may be interpreted as disturbed weighting of predictions and PEs.

### Active inference: control as a top-down prediction

To solve the computation problem in predictive coding, several algorithms have been proposed in the past, including the classic Kalman filter (Rao and Ballard, 1999), Bayesian belief propagation (Lee and Mumford, 2003), free energy minimization (Friston and Kiebel, 2009; Friston, 2010), backpropagation (Millidge et al., 2022b), and active inference (Kahl and Kopp, 2018; Parr et al., 2022; Millidge et al., 2022a). Specifically, active inference is an emerging theoretical framework that seeks to describe action and perception as inference-based computation (Pezzulo et al., 2015, 2018; Da Costa et al., 2020). Active inference employs actions to minimize PEs (Figure 2B), sharing some theoretical connections with control-as-inference and belief propagation (Friston et al., 2017; Millidge et al., 2020; Seymour and Mancini, 2020). Along a similar reasoning line, here we argue that active inference may serve as a generalized predictive coding algorithm for acute and chronic pain, where the cingulate-insula hub plays a critical role. First, the ACC has extensive connections with the motor cortex and spinal cord, connections that support the involvement of the ACC in motor control (Paus, 2001; Sheth et al., 2012). While the insula may be primarily responsible for inferring the latent pain state, the ACC may be involved in monitoring the action and feedback control (Fuchs et al., 2014). As a part of “perception-action loop”, motor control can be viewed as a special form of top-down sensory prediction. Further, ACC lesions may negatively affect action selection and adaptation (Brockett et al., 2020). Second, the discrepancy between the predicted and actual sensory feedback can be used as an indirect measure of “perceived controllability”, which is conceptually related to the “surprisal” in active inference (Smith R. et al., 2021, 2022). Consequently, low perceived controllability leads to maladaptive emotional and behavioral responses related to chronic pain. Real-time fMRI experiments have shown that healthy subjects and chronic pain patients could be trained to decrease activity in the ACC for pain relief, where lower pain ratings were related to greater control of neurofeedback (Chapin et al., 2012).

To perform hierarchical predictive belief propagation, multiple levels of predictions are sequentially computed. The lower level receives its next higher level’s prediction and evaluates it for its own bottom-up prediction in the next step (Figure 2A). The sensory prediction can influence both bottom-up (in the form of evidence for its last prediction from the next lower level) and top-down (in the form of a prediction by the next higher level) beliefs (Kahl and Kopp, 2018). One type of canonical neural networks with delayed Hebbian plasticity may prove to be a sufficient neural substrate to achieve active inference and control (Isomura et al., 2022; Isomura, 2022).

### Neural implementation of hierarchical predictive coding

How does the brain implement predictive coding? In an early proposal, Bastos and colleagues suggested that pyramidal cells at the superficial cortical layer—which are claimed to implement error units—are preferentially tuned to synchronization at the gamma band (30–90 Hz), whereas pyramidal cells at the deep layer—which implement prediction units—are tuned to synchronization in the slower alpha and beta bands (<30 Hz). Gamma-band synchronization may selectively increase the responsiveness of cortical error units without affecting the response of cortical prediction units that are tuned to signals at lower frequencies (Bastos et al., 2012). Additionally, an alternative proposal for neural implementation of predictive coding is to replace standard error units with dendritic error computation (Mikulasch et al., 2023), where the dendritic membrane potentials are integrated at the soma to form an error signal. Therefore, a spiking neuron can emit a spike when the somatic error potential grows too large, followed by a reduction in the overall error. In the hierarchical predictive coding model, the same error units can mediate bottom-up errors to update prediction units in the next level, as well as modulate top-down errors to neurons of the same level. The synaptic plasticity between error units and prediction units can be easily modulated by the classic Hebbian rule (Mikulasch et al., 2023). Despite the theoretical elegance, these two proposals are restricted to the implementation within different cortical layers (Shipp, 2016), and requires a much-needed update to accommodate predictive coding scenarios that are performed in different subdivisions of a cortical area, or in distributed neural circuits. Here we discuss a generalized version of this proposal in the context of pain processing.

First, at the cingulate-insula hub, different subdivisions of the cingulate cortex and insula cortex can implement the computation of PE or prediction separately. Take the insula cortex as an example, the PIC may contain the prediction units, whereas units from the AIC presumably either encode the error signals by its own, or receive predictions from the PIC, or even from the upstream structure. The prediction generated from the AIC may be further sent to the downstream structures (e.g., BLA) along the pain pathway. The intra-insula connectivity has a “closed-loop” structure, which may facilitate the intra-insula communications (Dionisio et al., 2019). Therefore, at the central hub, units with hierarchical connectivity can generate message passing between excitatory bottom-up and inhibitory top-down feedback. With proper excitatory and inhibitory interconnections, prediction and error units can emerge from biologically constrained recurrent neural networks (Ali et al., 2021).

Next, bottom-up and top-down signaling across hierarchical levels of pain circuitry is represented by mutually orthogonal neural oscillations. To date, frequency-specific neural oscillations have been reported in rodent and human pain studies, based on local field potentials, intracranial or scalp EEG recordings [see reviews in Ploner et al. (2017), Chen (2021), and Kim and David (2021)]. An important implication of the prediction coding theory is spectral asymmetry between neural signals representing predictions and neural signals representing PE, where the bottom-up or feedforward prediction signals are represented by higher frequency, and the top-down signals are represented by lower frequency through feedback (Arnal et al., 2011; Bastos et al., 2012, 2015; Michalareas et al., 2016; van Pelt et al., 2016; Chao et al., 2018). Additionally, higher frequency oscillations (>30 Hz, such as beta and gamma bands) are confined to a small neuronal space, whereas very large networks are recruited during slow oscillations (Buzsaki and Draguhn, 2004). In a recent human EEG study, Strube and colleagues investigated neural representations of predictions and PEs in heat and pain processing; they reported that the stimulus intensity expectation (“top-down signaling”) is associated with the alpha-to-beta band activity, whereas the PE (“bottom-up signaling”) is modulated by the gamma band activity (Strube et al., 2021). In another high-density ECoG recordings in monkeys, hierarchical predictive coding theory was validated for a large-scale cortical network spanning the auditory cortex, temporal cortex, and PFC. The lower- and higher-level PEs were identified in the early auditory cortex and anterior temporal cortex, respectively, whereas a prediction-update was sent from PFC back to temporal cortex; the PE and prediction-update was transmitted via gamma and alpha/beta oscillations, respectively (Chao et al., 2018).

Neural analysis of large-scale microelectrode array (MEA) and ECoG recordings revealed many traveling wave structures across a wide range of brain areas (for a review, see Muller et al., 2018). In the rodent hippocampus or nonhuman primate motor cortex, LFP-derived traveling wave patterns are found to be consistent with the traveling wave patterns derived from spiking activity (Patel et al., 2012; Takahashi et al., 2015). Furthermore, traveling waves may occur at multiple spatial scales. For instance, it was found in combined MEA and intracranial EEG recordings from epileptic patients that macro-scale traveling waves co-occurred with micro-scale traveling waves, which in turn were temporally locked to single unit spiking (Sreekumar et al., 2021). Human ECoG recordings have shown that theta and alpha oscillations tend to be spatially clustered with a traveling wave appearance propagating in a posterior-to-anterior direction (Zhang et al., 2018). Remarkably, recent human intracranial EEG data also showed that theta and beta oscillations are organized in the form of traveling waves along the anterior-posterior axis of the insula cortex, where the insular traveling waves at theta and beta frequency bands operate independently (Das et al., 2022). Importantly, traveling waves usually propagate from brain regions of higher-frequency oscillations to regions of lower-frequency oscillations (Zhang et al., 2018), reflecting an asymmetric information flow within the circuit hierarchy (Figure 2C). Therefore, neuronal oscillations can be hierarchically organized and carry independent information at different frequencies for intra-insula and inter-insula communications. Oscillatory multiplexing at various frequencies may provide a means for selective communication in the brain (Akam and Kullmann, 2014). Due to neural sampling and detecting issues, it is not unreasonable to believe that traveling waves are omnipresent across subcortical regions as well. Put together, hierarchically organized, multiscale traveling waves at multiple oscillatory frequencies provide a plausible brain mechanism to orchestrate and integrate bottom-up and top-down information across distributed pain circuits.

Finally, precision weighting is an important factor in predictive coding implementation at each level of hierarchical processing. One possible mechanism is through neuromodulators or neurotransmitters such as acetylcholine (ACh), norepinephrine (NE), and dopamine (DA), which have conceptual links to theories of attention and uncertainty (Friston, 2010). Several studies have shown that these neurotransmitters can regulate PEs and their precisions across different cortical hierarchies (Yu and Dayan, 2005; Moran et al., 2013). In the midbrain mesolimbic dopamine system, saliency-coding DA signaling responds to both appetitive and aversive stimuli (Becerra et al., 2001; Navratilova and Porreca, 2014), suggesting its modulation role in regulating pain-related PEs. Additionally, postsynaptic gain control at the cellular level has been implied in modulating the precision by changing the excitability of pyramidal neurons and neuronal time constants (Friston, 2005; Bastos et al., 2012). Finally, another possible mechanism is through fast synchronized presynaptic input that lowers effective postsynaptic membrane time constants and increases synchronous gain (Friston, 2010). The synchronous gain can shift neural activity from lower to higher frequencies (Auksztulewicz et al., 2017), such as increasing the power of gamma-band oscillations and decreasing the power of alpha oscillations, which has been reported as a correlate of predictability (Arnal et al., 2011; Brodski et al., 2015; Sedley et al., 2016). In the distributed pain circuits, despite limited direct experimental evidence, we envision that all three mechanisms can be independently or jointly implemented at various circuit nodes.

## Discussion and outlook

Thus far we have reviewed some experimental evidence and suggested how that can be fit into a conceptual hierarchical predictive coding framework. Within a distributed pain network, we argue that the ACC and insula serve as a central hub that mediate the information transfer or routing for PEs and predictions.

One of the implications of this framework is to formulate chronic pain as a result of abnormal predictive coding, in which the estimation of uncertainty of predictions or sensory inputs is systematically biased. For instance, the acetylcholine transmitter can modulate and regulate the sensory PEs, and cholinergic transmission can profoundly modify the perception of pain (Naser and Kuner, 2018). Therefore, neural pathways that involve medial septal (MS) cholinergic modulation to the rostral ACC can affect both perceptual and affective chronic pain behaviors (Jiang et al., 2018). Cholinergic signaling may also promote attention modulation that has an impact on nociception, pain, and even plasticity and learning, which have vital roles in pain chronification and maintenance (Apkarian et al., 2009).

Another important research direction is to apply this conceptual framework to make experimentally testable predictions. Any specific experimental hypotheses, once being rigorously tested, will improve current understanding of hierarchical predictive coding in distributed pain processing. Advances in high-density, large-scale electrophysiological and optical recordings (such as multifiber photometry) have become increasingly popular to simultaneously measure distributed cortical and subcortical brain areas (Chung et al., 2019; Juavinett et al., 2019; Sych et al., 2019; Steinmetz et al., 2021), which allows us to examine the neural coordination between pain upstream-downstream structures along the pathways. Second, optogenetic inactivation of ACC or insula nodes in animal models can reveal causal impact on neural representations and oscillations in the upstream or downstream pain circuits. Furthermore, optogenetic or spatiotemporally patterned stimulations that enhance or suppress specific neural oscillations (e.g., alpha or gamma bands) can test the specific role of top-down or bottom-up signaling in predictive coding. For instance, Additionally, virtual reality (VR) systems have provided a startlingly real simulation of the world that people can see, hear and touch, matching the real-world multisensory sensation of nociception and pain perception (Witttkopf et al., 2020; Trost et al., 2021). With combined VR and human EEG/fMRI studies, the hierarchical predictive coding framework may be extensively tested using the embodying prediction (Clark, 2013, 2016). For instance, competitive and precise sensory inputs can be introduced in the VR setting, and each input can be weighted by their precision (but such precise manipulation would be difficult in real-life experiments).

Finally, although we have focused on the “ACC-insula” saliency network as a predictive hub in this mini-review, several other brain areas such as the primary somatosensory cortex (SI), amygdala-hippocampus-NAc nodes in the limbic circuitry can also play relevant roles in hierarchical predictive coding. Our discussion here may serve as a starting point for pursuing similar questions at the computational, algorithmic, and implementation levels.

## Conclusion

In summary, predictive coding has become an increasingly powerful theory to unify large amount of seemingly different experimental data and understand the perception-action cycle in pain processing. Like any other research field, a theory is useful since it helps clarify and motivate thinking associated with observational studies (Levenstein et al., 2023). Similarly, algorithmic inference and high-level computational modeling may reveal insight into computational mechanisms of hierarchical predictive coding in pain studies (Alexander and Brown, 2018; Seymour and Mancini, 2020; Song et al., 2021). However, validation or refinement of this theory still requires further systematic investigations. We believe that at least two research directions may prove useful to help move forward towards that goal. First, combining optogenetics and large-scale and multisite electrophysiological neural recordings may enable us to uncover temporal activations of prediction-action pain circuits and delineate the causal link of neural circuits to pain-related behaviors. Development and applications of brain-machine interfaces (BMIs) will facilitate this effort (Zhang et al., 2021; Sun et al., 2022). Second, innovative designs of closed-loop human neuroimaging experiments may enable us to examine how the cingulate-insula hub dynamically changes its role in pain perception, motivation, and modulation.

## Author contributions

ZC conceived the ideas, supervised experiments, analyzed and interpreted the data, wrote the article, and acquired funding.
